# Integrative high-throughput studies to develop novel targets and drugs for the treatment of advanced prostate cancer

**DOI:** 10.1016/j.gendis.2025.101732

**Published:** 2025-06-23

**Authors:** Xuehui Li, Yanting Shen, Na Zhang, Dong Lu, Shuhua Ding, Fanchen Wu, Xiaowei Song, Xiangru Zhou, Shan Lin, Huan Xu, Zhong Wang, Fuwen Yuan

**Affiliations:** a Department of Urology and Andrology, Gongli Hospital of Shanghai Pudong New Area, Shanghai 200135, China; b Shanghai TCM-Integrated Hospital Affiliated with Shanghai University of Traditional Chinese Medicine, Shanghai 200082, China; c The Center for Cancer Research, School of Integrative Medicine, Shanghai University of Traditional Chinese Medicine, Shanghai 201203, China; d Department of Laboratory Medicine, Shanghai East Hospital, Tongji University School of Medicine, Shanghai 200123, China; e Institute of Interdisciplinary Integrative Medicine Research, Shanghai University of Traditional Chinese Medicine, Shanghai 201203, China; f Department of Urology, Shanghai Ninth People's Hospital, Shanghai Jiao Tong University School of Medicine, Shanghai 200011, China

**Keywords:** Advanced prostate cancer, CDC20, DTL, RB1/E2F1 axis, RRM2, Structure-based virtual screening

## Abstract

Androgen deprivation therapies targeting the androgen receptor (AR) signaling pathway are the primary treatment strategy for prostate cancer. However, these therapies often lead to castration resistance. Developing novel agents targeting AR-independent oncogenes is critical to address this challenge, particularly for advanced castration-resistant prostate cancer. This study identified three potential tumor drivers of advanced prostate cancer, including CDC20, DTL, and RRM2, through integrative bioinformatic screening that considered gene dependency using CRISPRi/RNAi database, clinical relevance, and experimental validation with CRISPR-Cas13-mediated gene ablation. Further mechanistic studies revealed that CDC20, DTL, and RRM2 were transcriptionally regulated by the RB1/E2F1 axis, mediating cell cycle progression in prostate cancer. Additionally, we identified novel agents targeting these candidates through virtual screening and drug-sensitive tests, utilizing our established small-molecule library. These agents exhibited superior anti-tumor efficacy compared with AR antagonists *in vitro*. Our study identified novel prostate cancer therapeutic targets independent of the AR signaling pathway and established a research paradigm for developing anti-tumor agents through integrative cancer bioinformatics and network pharmacology analysis.

## Introduction

Prostate cancer, one of the most prevalent cancers worldwide, is heavily dependent on the activation of the androgen receptor (AR) signaling pathway during its initiation and progression.^1^ However, agents targeting the AR signaling pathway only provide a moderate effect clinically. Tumors underwent lineage plasticity and developed resistance to androgen deprivation therapies due to the dysregulation of AR-independent genes, as we reviewed before,^2^ especially for neuroendocrine prostate cancer,^3^ in which stage, AR signaling is inactivated, and patients no longer respond to androgen deprivation therapies.^4^ Mechanistically, oncogenic mutations, amplifications, and tumor suppressor inactivation drive advanced prostate cancer progression in addition to the AR signaling pathway.^4^, ^5^, ^6^, ^7^ Therefore, uncovering and targeting core oncogenic drivers may offer new avenues for managing lethal prostate cancer. Notably, abnormal activity of cell-cycle machinery is essential for cancer progression and represents a driving force of tumorigenesis. The clinical success of cyclin-dependent kinase 4/6 (CDK4/6) inhibitors informs the feasibility of targeting cell-cycle components in breast cancer as an effective anti-tumor strategy.^8^^,^^9^

The retinoblastoma tumor suppressor gene RB1 is frequently inactivated in prostate cancer, primarily through deletion mutations and aberrant phosphorylation. RB1 dysregulation is a hallmark of prostate cancer, primarily recognized for its role in regulating the cell cycle through interactions with the early 2 factor (E2F) family of transcription factors, particularly E2F1.^10^, ^11^, ^12^ Notably, given the fundamental role of the E2F family in normal cellular processes, directly targeting the E2F family may not be desirable because of the potential for *in vivo* toxicity, although several agents, such as the pan-E2F inhibitor HLM006474 and the nucleoside analog LY101–4B, have been reported to suppress cancer growth *in vitro*.^13^^,^^14^ Instead, targeting downstream cancer-specific signatures of the RB1/E2F axis may represent a feasible strategy with higher specificity and fewer adverse effects. In this study, with unbiased integrative informatics analysis and molecular experimental validation, we surprisingly found that cell division cycle 20 (CDC20), denticleless E3 ubiquitin protein ligase (DTL), and ribonucleotide reductase M2 (RRM2), collectively referred to as CDRs, were specifically up-regulated in prostate cancer compared with normal prostate tissues and were further enhanced with prostate cancer progression as well as neuroendocrine prostate cancer (NEPC) lineage plasticity. Additionally, CDRs represent the prostate cancer-specific downstream effectors of the RB1/E2F axis, making them ideal therapeutic targets for prostate cancer in the context of cell cycle control.

Structure-based virtual screening is a computational technique used in drug discovery to predict the potential binding affinity between small molecules and target proteins.^15^ It helps prioritize compounds based on predicted binding affinity, which can focus experimental efforts on the most promising candidates, increasing the chances of identifying active molecules early in the drug discovery process.^16^^,^^17^ As a cost-effective, time-saving approach that enhances the efficiency and precision of drug discovery, especially when integrated with experimental techniques, virtual screening was employed in this study with our established small molecular library to screen agents targeting CDRs using our own established small molecular library, which includes diverse drug and natural product derivatives that were constructed via the advanced photochemical synthesis and late-stage modification of drugs and natural products. It should be noted that the addition of effective pharmacophores into the marketed drugs and bioactive molecules could enhance the bioactivity and druggability.^18^^,^^19^ Therefore, this library has superior drug similarity and structural diversity, indicating the strong potential to discover drug candidates from this library. More importantly, all of the synthesized molecules are easy to prepare on a gram scale, which could strongly support the in-depth pharmacological studies. Moreover, the leading compounds from the library are also highly likely to be optimized to improve their activity to find the final drug candidate. Here, with a structured-based virtual screening of agents targeting CDRs employing the molecular library, we identified a set of candidates that exhibited a potent tumor cell growth inhibition effect in prostate cancer, especially compounds, including Q199, XDD60, and A79, showed superior anti-tumor efficacy in advanced prostate cancer cell models compared with the clinical prevalent AR antagonist enzalutamide.

## Materials and methods

### Cell source and culture conditions

Prostate cancer cell lines C4-2B and PC3 were purchased from SUNNCELL (SNL-161) and the National Collection of Authenticated Cell Cultures Bank (TCHu158), respectively. All cells were cultured within RPMI 1640 (Macgene, CM10041) supplemented with 10% fetal bovine serum (Gibco, 10270-106). Cells were maintained within a humidified incubator at 37 °C in the presence of 5% CO_2_. Fresh medium was replaced every two days, and cells were dissociated for passaging when the confluence reached 80%–90%.

### CRISPR-Cas13-mediated target silencing

Three guide RNAs (gRNA) for each target, including RB1, E2F1, CDC20, RRM2, and DTL, were designed, generated, and cloned into CRSIPR-Cas13 corresponding gRNA backbone (Addgene, 109053). The gRNA plasmid and CRISPR-Cas13 plasmid (Addgene, 109049) were co-transfected into prostate cancer cells with Lipomaster 3000 Transfection Reagent (Vazyme, TL301-01) as we described previously.^20^ The target gene silencing efficiency was determined with quantitative real-time PCR (qRT-PCR). The gRNA sequences are listed in Table S1.

### qRT-PCR

To determine the mRNA expression of the interested genes, total RNA was extracted using the FastPure Isolation Kit (Vazyme RC112-01, China). The first strand cDNA was reversed with the HiScript II Q RT SuperMix (Vazyme, RC223-01) and amplified with the ChamQ Universal SYBR qPCR Master Mix (Vazyme, Q711-02). The indicated gene expression was analyzed as we described before^20^ in the LightCycler 480 II machine (Roche). The primers used are listed in Table S2.

### Western blotting

The protein expression of cyclin D1 (CCND1), cyclin-dependent kinase 1 (CDK1), p-CDK1, and actin-beta (ACTB) was evaluated using western blotting assays as we described previously.^21^ Briefly, cells collected from 6-well plates were lysed in 0.2 mL of RIPA lysis buffer (Beyotime P0013B, China) with protease and phosphatase inhibitors (Beyotime P1045, China) and applied for protein separation in SDS-PAGE gel. The primary antibodies used in this study were as follows: p-CDK1 (Santa Cruz Biotechnology sc-136,014, 1:500), CDK1 (ABclonal A12414, 1:1000), CCND1 (ABclonal A19038, 1:1000), and ACTB (Servicebio GB15001, 1:2000).

### Cell viability assays

Cell viability was determined as described before.^20^ Briefly, cells were planted in 96-well plates, the specified doses of compounds were added after 24 h of incubation, and the cells were cultured for 48 h. The Cell Counting Kit-8 (CCK-8) reagent (GlpBio, GK10001) was then added and incubated for another 2 h. The absorption at 450 nm was measured using a 96-well plate reader (TECAN Spark, Switzerland).

### ChIP-qPCR

Chromatin immunoprecipitation (ChIP) assays were performed as we described previously.^22^ Briefly, cells were fixed with 1% formaldehyde for 10 min, and chromatin was collected, sonicated, diluted, and immunoprecipitated with specific antibodies. Protein A-Sepharose beads were added and incubated for another 1 h with rotation. The beads were then washed sequentially in TSE I, TSE II, and buffer III. Chromatin complexes were eluted with an elution buffer. DNA fragments were purified with a PCR purification kit (Yeasen, 19106ES08) for qPCR analysis. Primers used are listed in Table S3.

### CRISPR-Cas9 gene dependency analysis

The gene effects on different prostate cancer cell models (DepMap Public 24Q2+Score, Chronos) were obtained from the DepMap Portal (https://depmap.org/portal/). The influence of each gene on the different prostate cancer cell models was analyzed individually.

### Public data access of prostate cancer cohorts

The gene expression data for prostate cancer tissues and adjacent normal prostate tissues, along with the corresponding clinical data of the patients, including tumor stage, Gleason score, NEPC feature, and patient prognosis, were obtained from the Genomic Data Commons (GDC) Data Portal (https://portal.gdc.cancer.gov/), cBioPortal (https://www.cbioportal.org/) and GEPIA2 (http://gepia2.cancer-pku.cn/#index). The differential expression gene heatmap and survival analysis were conducted using GraphPad Prism 10 and RStudio.

### Pathway enrichment analysis of CDRs coexpressed genes

The CDRs coexpressed genes from the ADPC patients cohort (TCGA-PRAD) and CRPC cohorts^23^^,^^24^ were retrieved from cBioPortal (https://www.cbioportal.org/), and the overlapped CDRs coexpressed gene signature was then uploaded to the DAVID bioinformatics platform (https://davidbioinformatics.nih.gov/tools.jsp) for pathway enrichment analysis.

### Small molecular library generation

The small library was supported by the group of Yu Zhang.^25^, ^26^, ^27^ They have developed several efficient photochemical strategies to construct novel molecules, including plenty of drug derivatives and natural product analogues. The synthesized molecules have been classified by their key scaffolds, including several prevalent drug motifs. The details of the included units are shown in Table S4.

### Structure-based virtual screening

Small molecules used in virtual screening were from an in-house natural product-derived compound database containing over 1300 compounds. The structures of all the molecules were then energy-minimized by the Molecular Operating Environment (MOE) software, using the MMFF94 force field with the gradient convergence set to 0.1 kcal/mol. The crystal structures of the CDC20 (PDB id: 6q6g) and RRM2 (PDB id: 3olj) were downloaded from the RCSB Protein Data Bank (PDB, https://www.rcsb.org/). As there was no crystal structure for DTL, its predictive 3D structure from AlphaFold2 (https://alphafold.ebi.ac.uk/) was used for virtual screening. The water molecules farther than 4.5 Å from the above protein receptors were removed. Then, all the protein structures were prepared by the built-in MOE structure preparation and Protonate3D software tools using the default parameters. The ligand binding site for CDC20 was set in the pocket near the residue Val200. For RRM2, the site was set around the residue Arg330. The binding pocket of DTL was generated by the SiteFinder module in MOE. The placement method was set to Triangle Matcher with the London dG scoring function, while the refinement score algorithm was set to GBVI/WSA dG. Then, all the small molecules were docked into each protein binding pocket. Finally, the top 50 compounds with the highest scores for each protein were selected for further *in vitro* experiments.

### Statistical analysis

Statistical significance was evaluated using two-sided unpaired *t*-tests. In the figures with bar graphs, results were expressed as mean ± standard deviation, and *p* ≤ 0.05 was considered statistically significant (∗∗*p* ≤ 0.01, ∗*p* ≤ 0.05).

## Results

### Integrative screening identified prostate cancer-specific gene signatures

**Figure 1:**
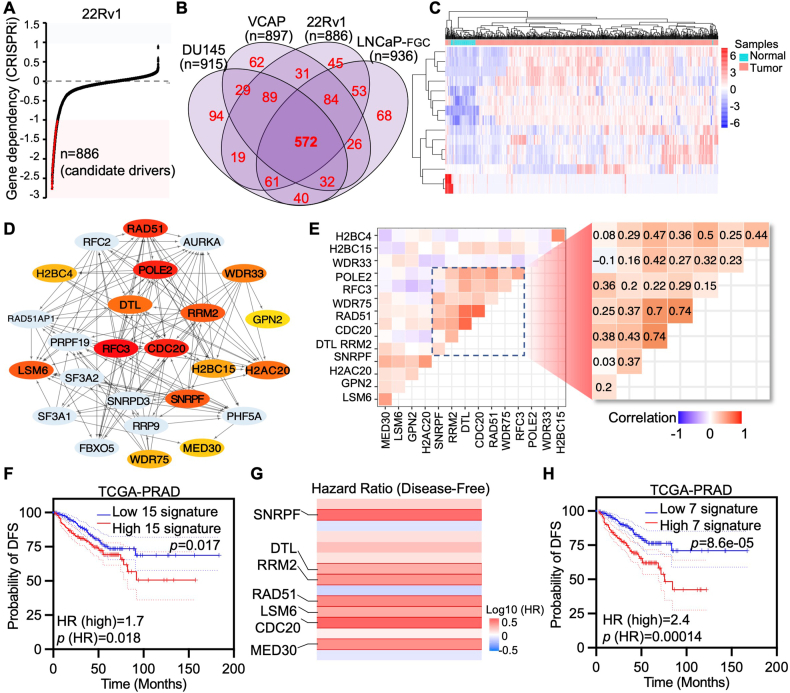
Integrative analysis revealed a prostate cancer-specific signature comprised of seven genes. **(A)** Visualization of representative gene dependency in 22Rv1 cells with CRISPR-Cas9 screening. **(B)** The Venn diagram indicates the genes whose ablation significantly suppresses prostate cancer cell growth in different prostate cancer cell models. **(C)** The heatmap shows the expression of genes differentially expressed in prostate cancer compared with normal prostate tissues in the TCGA-PRAD cohort. **(D, E)** Protein–protein interaction string (D) and expression correlation (E) of genes that were both dysregulated in prostate cancer and suppressed cell growth after being silenced in the TCGA-PRAD cohort. **(F, G)** Survival analysis of the gene signature comprised of 15 genes (F) as well as individuals (G) with the TCGA-PRAD prostate cancer cohort. **(H)** Survival analysis of the core gene signature comprised of 7 genes with the TCGA-PRAD prostate cancer cohort.

To identify oncogenic genes that may serve as prostate cancer-specific targets, we conducted a genome-wide screening based on two criteria: 1) ablation of the gene suppresses cancer cell growth *in vitro*, and ii) its expression is elevated in cancer compared with normal prostate tissues and correlates with prostate cancer prognosis. Results showed that a total of 572 genes were identified whose ablation significantly suppressed cell viability across four prostate cancer cell lines (Fig. 1A, B; Fig. S1A–S1C). Further analysis of these genes in the TCGA-PRAD dataset revealed that 60 genes were dysregulated in cancer tissues compared with normal prostate tissues, with 15 genes showing significant up-regulation in cancer (Fig. 1C; Fig. S1D). STRING analysis of protein–protein interactions revealed significant correlations among these genes, and survival analysis of the 15-gene signature indicated a worse patient prognosis (Fig. 1D–F; Fig. S2). Notably, a core signature comprising seven genes, including small nuclear ribonucleoprotein polypeptide F (SNRPF), replication factor C subunit 3 (RFC3), WD repeat domain 75 (WDR75), RAD51 recombinase (RAD51), CDC20, DTL, and RRM2, showed a strong correlation across multiple prostate cancer cohorts at different progression stages. This core signature demonstrated greater prognostic significance compared with the 15-gene signature, with enhanced expression of these genes being more strongly associated with worse disease-free survival (Fig. 1F–H).

### CDRs linking to prostate cancer prognosis and NEPC lineage plasticity

**Figure 2:**
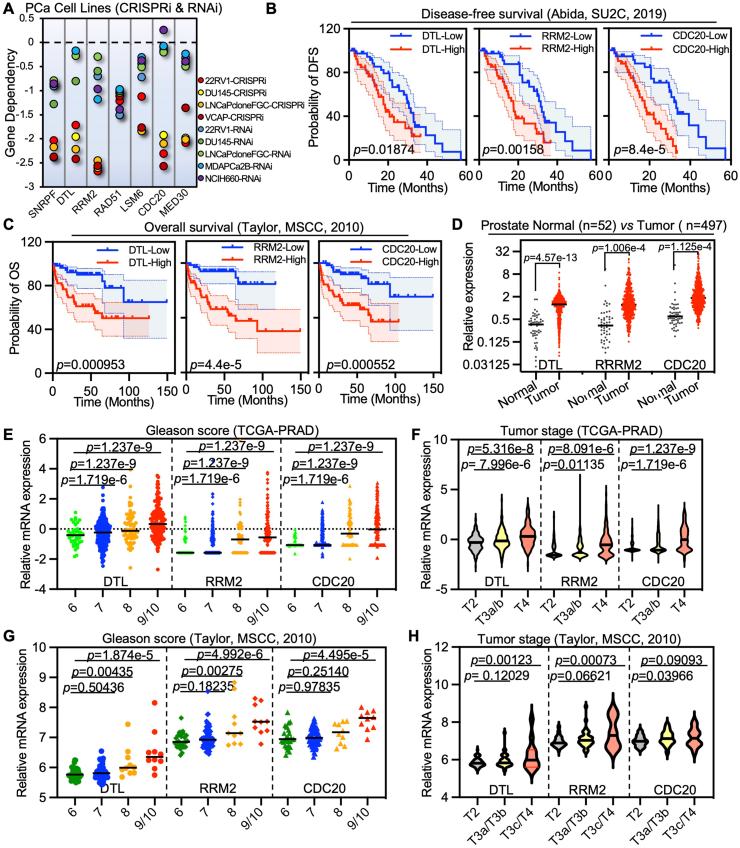
Enhanced CDRs correlated with prostate cancer prognosis. **(A)** Visualization of core genes' dependency in different prostate cancer cells with CRISPR-Cas9 and siRNA screening. **(B, C)** Survival analysis of CDRs in different CRPC patient cohorts. **(D)** The relative expression of CDRs in normal prostate tissues and prostate cancer tissues in the TCGA-PRAD cohort. Gleason score and tumor stage correlation analysis of CDRs in different prostate cancer patient cohorts. All prostate patient cohorts were indicated in the corresponding panels. CDRs refers to CDC20 (cell division cycle 20), DTL (denticleless E3 ubiquitin protein ligase), and RRM2 (ribonucleotide reductase M2).

We next analyze the role of each specific gene in the core signature associated with prostate cancer. The impact of the seven genes included in the core signature was evaluated in six prostate cancer cell lines using CRISPR-Cas9 or siRNA-mediated gene silencing, which demonstrated a general inhibition of cell growth. Notably, the knockdown of CDRs resulted in a significant suppression of tumor cell growth (Fig. 2A). Further analysis of the expression of each gene concerning prostate cancer survival across multiple cohorts revealed a significant correlation between CDRs and patient survival (Fig. 2B, C; Fig. S3). Additionally, the expression of CDRs was significantly up-regulated in prostate cancer alongside prostate cancer progression, either determined by Gleason score or tumor stage across various cohorts (Fig. 2D–H; Fig. S4). Collectively, these data indicate the oncogenic potential of CDRs in prostate cancer.

**Figure 3:**
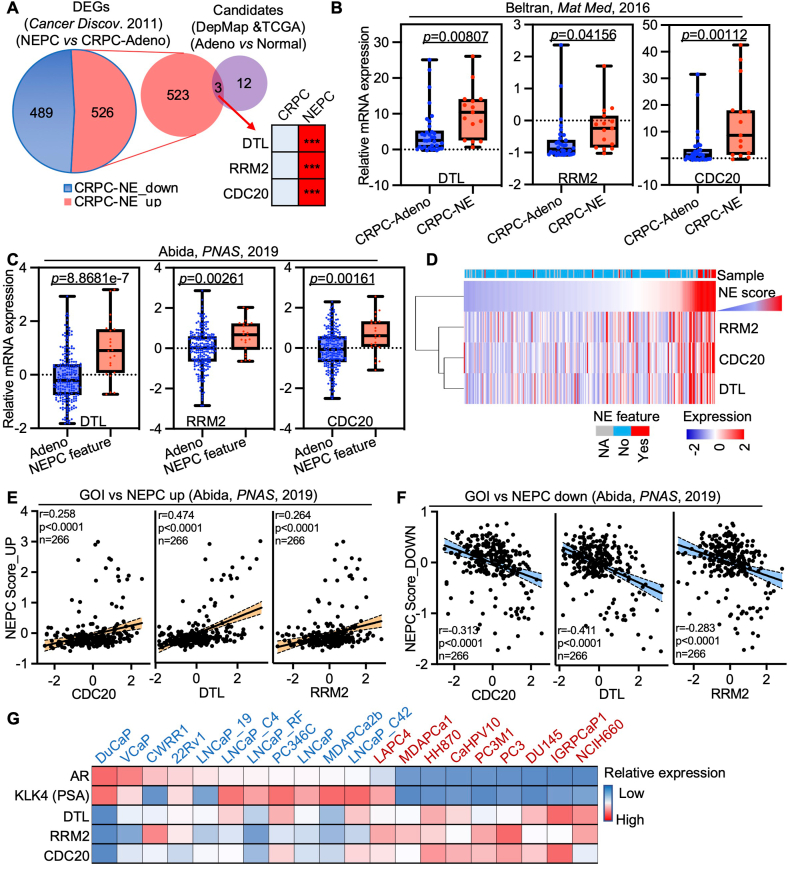
CDRs linked with neuroendocrine features in prostate cancer cohorts. **(A)** The Venn diagram illustrates the genes specifically enhanced in prostate cancer compared with normal prostate tissues and NEPC compared with adenocarcinoma. **(B, C)** The relative expression of CDRs in NEPC compared with adenocarcinoma in different prostate cancer cohorts. **(D)** Correlation analysis of CDRs with NE score. The red indicates the higher NE score. **(E, F)** Pearson correlation analysis of CDRs with NE score CRPC cohort. **(G)** The heatmap indicates the relative expression of CDRs and AR, as well as KLK3, a transcription activity indication of AR. CDRs refers to CDC20 (cell division cycle 20), DTL (denticleless E3 ubiquitin protein ligase), and RRM2 (ribonucleotide reductase M2). NEPC, neuroendocrine prostate cancer; NE, neuroendocrine; AR, androgen receptor; KLK3, kallikrein-related peptidase 3.

NEPC, characterized by a diminished AR signaling pathway, is a lethal stage of prostate cancer that does not respond to androgen deprivation therapies, the primary treatment strategy for both ADPC and CRPC. Patients at this stage often succumb quickly due to the absence of standard therapies. Given that our study demonstrated a correlation between CDRs and prostate cancer prognosis, prompting us to investigate whether CDRs could serve as potential treatment targets for NEPC. Surprisingly, CDRs were significantly overexpressed in prostate cancer tissues compared with normal prostate tissues, and in NEPC compared with CRPC across multiple prostate cancer cohorts (Fig. 3A–C). Furthermore, CDR expression was positively correlated with NEPC scores and NEPC features (Fig. 3D–F; Fig. S5). This finding underscores the relationship between CDRs and NEPC lineage plasticity. Notably, the expression levels of CDRs and AR in 19 prostate cancer cell lines demonstrated a significant decrease in CDRs in cells exhibiting lower levels of AR (Fig. 3G). These data collectively indicate that CDRs are associated with prostate cancer progression and NEPC characteristics.

### CDRs contribute to prostate cancer survival by regulating cell proliferation

**Figure 4:**
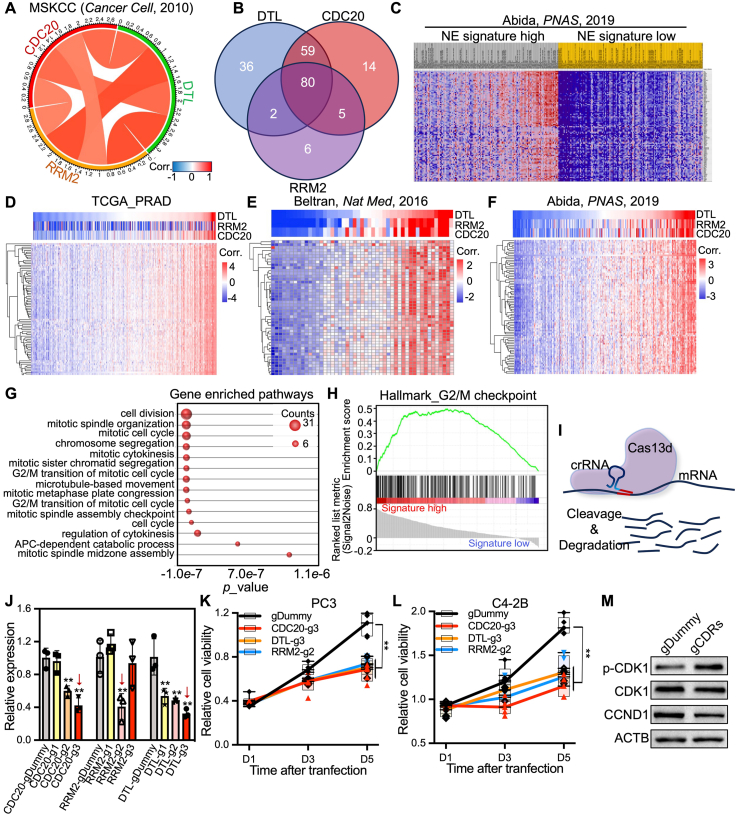
CDRs ablation suppressed prostate cancer cell proliferation. **(A)** The circuit diagram illustrates the correlation among CDRs co-expressed gene sets. **(B)** Venn analysis of the overlap of CDRs co-expressed genes. **(C)** The heatmap shows the relative expression of CDRs overlapped co-expressed genes in patients with NE signature high and low groups. **(D**–**F)** The heatmap illustrates the correlation of CDRs co-expressed genes with CDRs in ADPC (D) and CRPC (E, F) patient cohorts. **(G, H)** KEGG pathway analysis (G) and GSEA analysis (H) of enriched biological processes of CDRs and CDRs co-expressed genes. **(I, J)** The diagram illustrates the working model of CRISPR-Cas13 for RNA silencing and knockdown efficiency of CDRs with the corresponding gRNAs. **(K, L)** Cell growth assays indicate the impact of CDRs knockdown on the viability of different prostate cancer cells. **(M)** Western blotting analysis of the expression of cell cycle-regulated genes, including CCND1, CDK1, and p-CDK1, after CDRs knockdown. ∗∗*p* < 0.01. CDRs refers to CDC20 (cell division cycle 20), DTL (denticleless E3 ubiquitin protein ligase), and RRM2 (ribonucleotide reductase M2). NE, neuroendocrine; KEGG, Kyoto Encyclopedia of Genes and Genomes; GSEA, gene set enrichment analysis; CCND1, cyclin D1; CDK1, cyclin-dependent kinase 1.

We next wondered about the roles and mechanisms of highly expressed CDRs in advanced prostate cancer. We retrieved the co-expressed gene list of each of CDC20, RRM2, and DTL across ADPC, CRPC, and NEPC cohorts, which showed 175, 92, and 159 genes significantly co-expressed with CDRs at different prostate cancer stages, respectively (Fig. 4A–F; Fig. S6). Surprisingly, we observed a significant overlap and correlation among the gene sets associated with CDC20, RRM2, and DTL (Fig. 4A, B). Pathway analysis and gene set enrichment analysis (GSEA) of CDRs co-regulated gene set revealed a significant enrichment of cell proliferation-related biological processes, especially cell cycle regulation-related pathways, such as G2/M checkpoint, cell division, and cell cycle (Fig. 4G, H). To further confirm the role of CDRs in prostate cancer cell growth, we ablated the endogenous expression of CDC20, RRM2, and DTL with cutting-edge RNA-targeted CRISPR-Cas13 (Fig. 4I, J). CCK-8 assays were performed to assess the impact of CDRs silencing on cell growth, which demonstrated a significant tumor cell growth inhibition effect in both C4-2 and PC3 cells regardless of the AR signaling activity (Fig. 4K, L). We next conducted a western blotting analysis of cell cycle-related genes, including CCND1, CDK1, and p-CDK1, and results showed that CDRs knockdown significantly increased p-CDK1, which was reported to induce G2/M cell cycle arrest^28^ (Fig. 4M). These studies suggest that targeting CDRs may represent a viable strategy to treat lethal-stage prostate cancer, such as CRPC and NEPC. It is worth noting that claudin-3 (CLDN3) and claudin-4 (CLDN4) have been reported to be implicated in dictating malignant progression in prostate cancer, especially in neuroendocrine tumors similar to CDRs.^29^, ^30^, ^31^ However, no significant correlations between CDRs or E2F1 and CLDN3/CLDN4 expression were observed (Fig. S7A, B), and CDRs depletion did not statistically affect CLDN3 or CLDN4 expression (Fig. S7C), indicating that CDRs and CLDN3/CLDN4 likely promote tumor progression through distinct molecular pathways.

### RB1/E2F1 axis governs the transcription of CDRs

**Figure 5:**
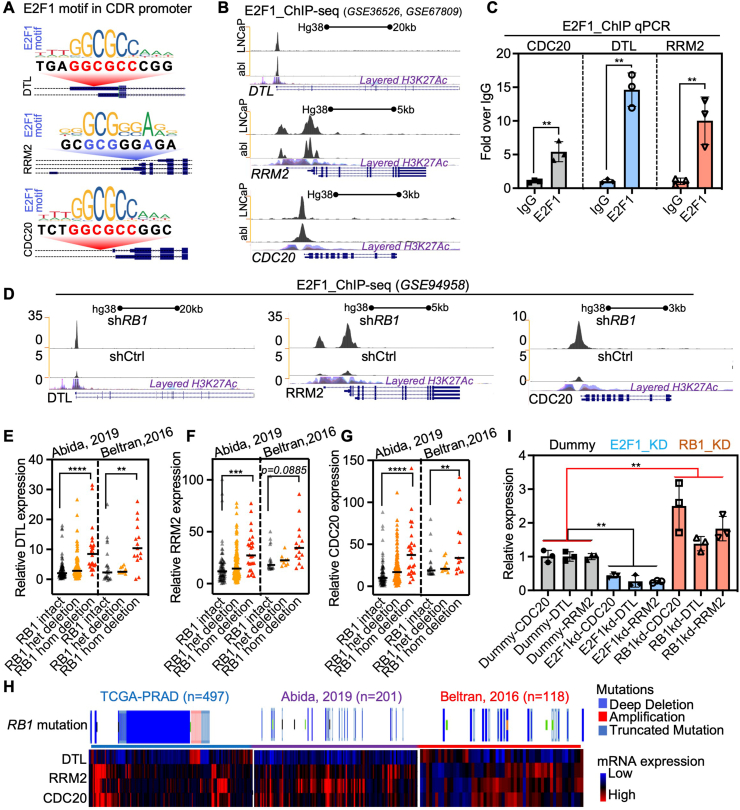
CDRs were transcriptionally regulated by the RB1/E2F1 axis in prostate cancer. **(A)** The diagrams show the canonical E2F1 motif location within the promoter of CDRs. **(B)** ChIP-sequencing E2F1 enrichment peak within the promoter of CDRs. **(C)** The relative enrichment of E2F1 within the promoter of CDRs was determined using standard ChIP-qPCR. **(D)** ChIP-sequencing peaks show the relative enrichment of E2F1 in CDRs' promoters after *RB1* is known. **(E**–**G)** The relative expression of CDRs in CRPC patients with different *RB1* deletion status. **(H)** CDRs expression in patients with *RB1* deletion mutations from different prostate cancer cohorts. **(I)** qRT-PCR detected the relative expression of CDRs after RB1 or E2F1 knockdown with CRISPR-Cas13. ∗∗*p* < 0.01, ∗∗∗*p* < 0.001, and ∗∗∗∗*p* < 0.0001. CDRs refers to CDC20 (cell division cycle 20), DTL (denticleless E3 ubiquitin protein ligase), and RRM2 (ribonucleotide reductase M2). RB1, retinoblastoma tumor suppressor 1; E2F1, early 2 factor 1; ChIP, chromatin immunoprecipitation; qRT-PCR, quantitative real-time PCR.

Given that CDRs, as well as CDRs co-expressed genes, are significantly correlated both in expression patterns and biological processes (Fig. 4A–F), we hypothesized that CDC20, RRM2, and DTL may be regulated by the same upstream molecules and pathways. We subsequently analyzed the promoter regions of CDRs and identified canonical E2F1 binding motifs within all CDR promoters (Fig. 5A). Further peak visualization of ChIP-sequencing data in both prostate cancer cell LNCaP and abl showed significant binding enrichment of E2F1 within the promoters of CDRs (Fig. 5B), which was further validated with ChIP-qPCR experiments in prostate cancer cells (Fig. 5C). Additionally, ChIP-qPCR performed with a specific antibody recognizing E2F1 indicated enrichment of E2F1 in the promoters of CDRs. Moreover, the knockdown of *RB1*, an E2F1 transcriptional activity regulator that is frequently mutated in advanced prostate cancer and suppresses cancer lineage plasticity, metastasis, and androgen deprivation therapy resistance,^32^ significantly decreased the enrichment of E2F1 within the promoters of CDRs in prostate cancer cells (Fig. 5D). Notably, in three independent cohorts of advanced prostate cancer, *RB1* deletion mutations significantly up-regulated the expression of CDRs (Fig. 5E–H; Fig. S8). Knockdown of the expression of either *E2F1* or *RB1* with CRISPR-Cas13 mediated transcriptional perturbation of CDRs (Fig. 5I). Collectively, these results demonstrate that the RB1/E2F1 axis mediates the enhanced expression of CDRs in advanced prostate cancer.

### Virtual screening identified agents targeting CDRs

**Figure 6:**
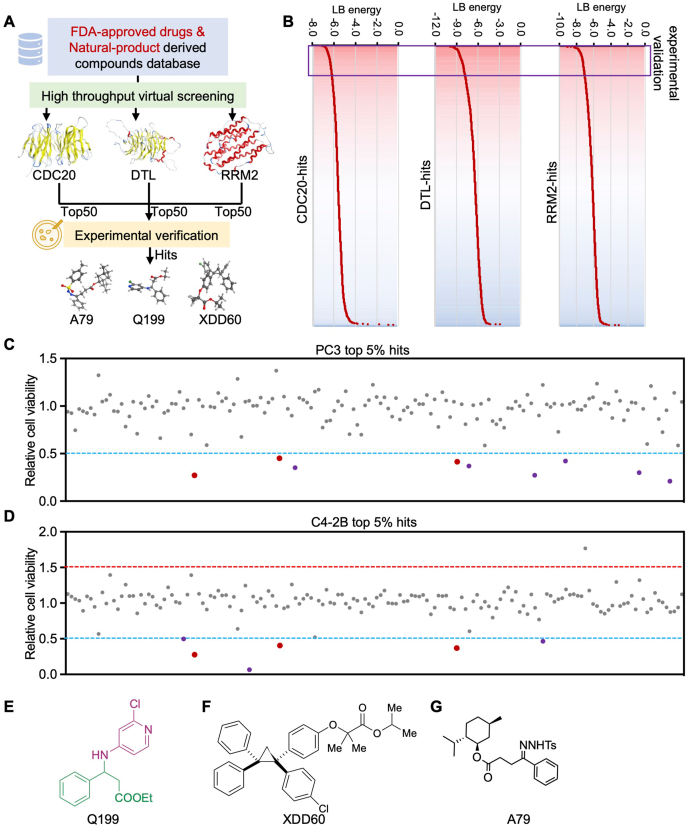
Virtual screening identified compounds that suppressed advanced prostate cancer. **(A)** The diagram illustrates structure-based virtual screening strategies for CDRs-targeted compounds. **(B)** The heatmap shows the binding affinity of compounds with CDRs. The red indicated a higher binding affinity of compounds with the corresponding compounds. **(C, D)** Cell viability assays were used to determine the tumor suppressive effect of compounds with high CDRs-binding affinity in different prostate cancer cell models. **(E**–**G)** The 2D structure of Q199, XDD60, and A79, which exhibits the most significant anti-tumor efficacy in prostate cancer cell models. CDRs refers to CDC20 (cell division cycle 20), DTL (denticleless E3 ubiquitin protein ligase), and RRM2 (ribonucleotide reductase M2).

Given the pivotal role of CDRs in prostate cancer progression, we propose that small molecules targeting CDRs may serve as a viable strategy for treating lethal prostate cancer. To identify potentially active compounds, we employed structure-based virtual screening using our established molecular library consisting of more than 1200 molecules belonging to ten types with different skeletons (Table 1). High-throughput virtual screening was conducted for each CDR protein, and compounds were ranked based on docking scores, which represent the binding affinity between the compound and the protein receptor (Fig. 6A). Lower docking scores indicate stronger affinity. From this screening, the top 50 compounds for each protein were selected for further experimental validation in advanced prostate cancer cell models (Fig. 6B). Cell growth assays revealed that nine compounds suppressed PC3 cell growth, while six compounds inhibited C4-2B cell growth (Fig. 6C, D), among which, Q199, XDD60, and A79 belong to various categories with diverse pharmacophores, demonstrated potent cancer cell growth suppression in both cell models (Fig. 6C–G), highlighting their potential as anti-tumor agents targeting CDRs in prostate cancer.

**Table 1:** Rapid construction of small-molecule library employed for virtual screening.

Categories	Numbers	Core structure	Representative drugs
Hydrazones	288		
Olefins	79		
N-heterocycle	409		
Three-membered structure	61		
Ethers	98		
Flavanones	116		
Amines	97		
Borides	10		
Fluorenes	62		
Ezetimibe- derivatives	24		
Others	12	–	–

### Small molecules targeting CDRs exhibit superior anti-tumor efficacy in prostate cancer than enzalutamide *in vitro*

**Figure 7:**
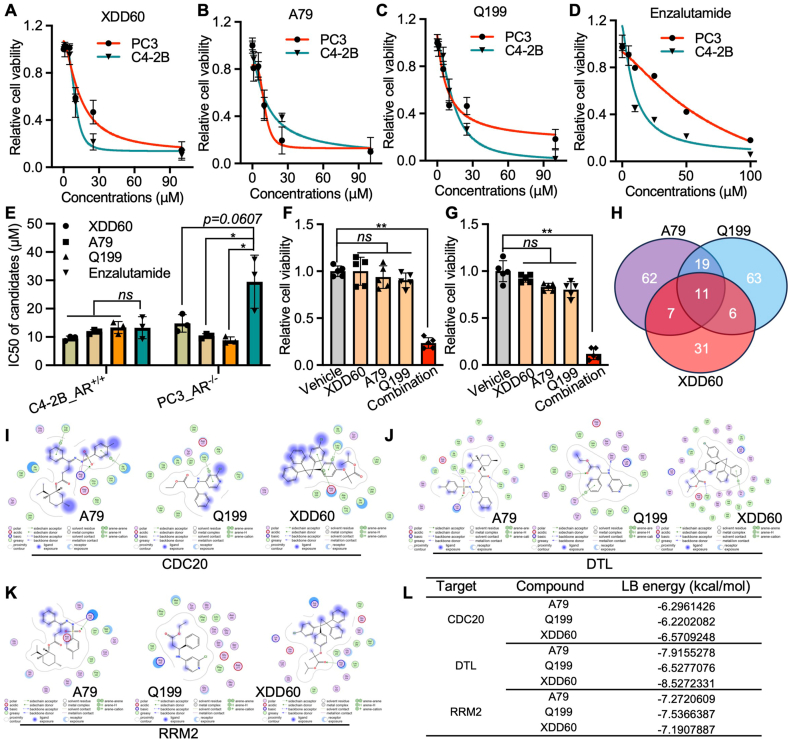
Compounds targeting CDRs exhibited superior anti-tumor efficacy compared with AR antagonists. **(A**–**E)** The tumor cell growth inhibition effects of different dosages of Q199, XDD60, and A79, as well as enzalutamide, were determined with CCK-8 assays (A–D), and the IC_50_ of each agent was calculated with three independent experiments (E). **(F, G)** The histograms show the relative cell viability after being treated with 5 M of Q199, XDD60, or A79 alone, or a combination. **(H)** The Venn diagram shows the overlap of Q199, XDD60, and A79 potential targets predicted with SwissTargetPrediction (http://swisstargetprediction.ch/). Molecular docking shows the binding of CDRs with Q199, XDD60, and A79. **(I)** The lowest binding (LB) affinity of CDRs with Q199, XDD60, and A79. ns, not significant. ∗∗*p* < 0.01. CDRs refers to CDC20 (cell division cycle 20), DTL (denticleless E3 ubiquitin protein ligase), and RRM2 (ribonucleotide reductase M2). AR, androgen receptor.

To further evaluate the anti-tumor efficacy of these compounds, we determined their IC_50_ values in prostate cancer cells and compared them to enzalutamide, a widely used AR antagonist. Results showed that the IC_50_ values of Q199, XDD60, and A79 were comparable to or even lower than those of enzalutamide, particularly in AR-inactive prostate cancer cell models (Fig. 7A–E). Given that CDRs share common biological processes, we hypothesized that combining low doses of Q199, XDD60, and A79 might yield a more significant tumor growth inhibition effect than individual compounds. As shown in Figure 7F and G, a combination of 5 μM of each compound, which alone had no significant tumor suppression effect, markedly inhibited cell growth. Target prediction analysis indicated that these compounds have distinct targets (Fig. 7H), which is not surprising given that these compounds belong to various categories with diverse pharmacophores. This suggests that the combination may have lower potential toxicity *in vivo* due to minimal overlap in off-target effects.

Molecular docking studies further confirmed the strong binding affinity of these compounds with all CDRs (Fig. 7I–L; Fig. S9). For example, Q199 exhibited a docking score of −6.22 kcal/mol when complexed with CDC20, forming an arene–H interaction with Leu176 and stabilizing its binding through hydrophobic interactions with the protein receptor (Fig. S9). These findings collectively underscore the potential of Q199, XDD60, and A79 in managing lethal prostate cancer. However, further studies are needed to evaluate their anti-tumor efficacy and potential toxicity *in vivo*.

## Discussion

Through integrative studies, including CRISPR-Cas9i screening across various prostate cancer cell models, analysis of clinical cancer-specific expression, relevance to patient prognosis, and *in vitro* loss-of-function validation, we identified three cancer-specific targets: CDC20, RRM2, and DTL. While previous studies have highlighted the oncogenic roles of CDC20 and RRM2 in prostate cancer,^33^, ^34^, ^35^, ^36^ our work further demonstrates that DTL plays a comparable tumor-driving role in prostate cancer compared with CDC20 and RRM2. Importantly, through virtual screening using our established small-molecule library (Table 1), we identified a group of compounds with high binding affinity to CDRs (Fig. 6). Among these, Q199, XDD60, and A79 exhibited comparable or even superior tumor growth inhibition efficacy compared with enzalutamide (Fig. 7A–E), a clinically prevalent AR antagonist. This study establishes a research paradigm for identifying novel disease-specific targets and corresponding drugs through integrative CRISPR-Cas9i gene dependency screening, high-throughput clinical database analysis, small-molecule virtual screening, and experimental validation.

To elucidate the role and mechanisms of CDRs in prostate cancer, we investigated both their downstream pathways and upstream regulators. We discovered that all three targets—CDC20, RRM2, and DTL—converge on the same biological pathways, particularly those involved in cell cycle regulation (Fig. 4A–H). Furthermore, we identified canonical E2F1 binding motifs within the promoters of CDRs, and CRISPR-Cas13-mediated ablation of RB1/E2F1 abolished CDR transcription (Fig. 5). The RB1/E2F1 axis is closely associated with prostate cancer neuroendocrine differentiation,^32^^,^^37^ and its dysregulation is linked to uncontrolled cell cycle progression during cancer development.^14^^,^^38^ These findings, supported by both previous studies and our data from clinical cohorts and *in vitro* experiments, suggest that dysregulation of the RB1/E2F1 axis in advanced prostate cancer may drive lethal disease progression by regulating CDRs.

Although targeting RB1 loss or E2F1 has long been considered a promising strategy for cancer therapy,^14^^,^^39^ currently, there are no agents that directly target RB1/E2F1 in the market due to their undruggable characteristics as well as their general roles in biological processes in the healthy body. Alternatively, we reported here that targeting the downstream core cancer drivers (CDRs) of RB1/E2F1, either through gene silencing using specific CRISPR-Cas13 (Fig. 4I–L) or with small molecular inhibitors (Fig. 7A–G), may achieve anti-tumor goals. Notably, a combination of Q199, XDD60, and A79 at relatively low dosages significantly suppressed prostate cancer cell growth (Fig. 7F, G). This approach leverages compounds that target different cancer drivers within the same pathway, potentially reducing toxicity due to their distinct off-target effects. However, further *in vivo* studies are needed to validate this hypothesis and assess potential toxicity.

This study has several limitations. First, our screening strategy, which relied on mRNA expression analysis, may have missed oncogenes whose pro-tumor functions are driven by mutations or protein-level alterations, such as translation, modifications, or degradation. Expanding proteomics databases in prostate cancer cohorts, including protein expression and modification data, could significantly enhance the identification of novel anti-tumor targets. Second, while we identified several therapeutic agents exhibiting high binding affinity to CDRs and promising suppression of prostate cancer growth *in vitro*, we were unable to evaluate their anti-tumor efficacy and safety *in vivo* due to the current lack of sufficient quantities of these compounds. Validating their anti-tumor potential and toxicity profiles *in vivo* will be prioritized in subsequent phases of our research. Additionally, we are currently optimizing these candidates to improve their binding affinity and bioavailability.

## CRediT authorship contribution statement

**Xuehui Li:** Writing – original draft, Visualization, Resources, Methodology, Investigation, Formal analysis, Data curation. **Yanting Shen:** Writing – review & editing, Validation, Resources, Methodology, Investigation, Formal analysis. **Na Zhang:** Writing – review & editing, Validation, Resources, Investigation, Formal analysis, Data curation. **Dong Lu:** Writing – review & editing, Visualization, Validation, Software, Resources, Methodology, Formal analysis, Data curation. **Shuhua Ding:** Resources, Formal analysis, Data curation. **Fanchen Wu:** Visualization, Formal analysis. **Xiaowei Song:** Validation, Formal analysis. **Xiangru Zhou:** Visualization. **Shan Lin:** Supervision, Resources. **Huan Xu:** Writing – review & editing, Supervision, Software. **Zhong Wang:** Supervision, Software, Funding acquisition. **Fuwen Yuan:** Writing – original draft, Supervision, Resources, Project administration, Funding acquisition, Conceptualization.

## Funding

This study was supported by the 10.13039/501100001809National Natural Science Foundation of China (No. 82473185, 82202922) and the Key Discipline Development Initiative of the Shanghai Health System (China) (No. 2024ZDXK0043).

## Conflict of interests

The authors declared no conflict of interests.
