# Supplementary material for: Integrative high-throughput studies to develop novel targets and drugs for the treatment of advanced prostate cancer
Source: Genes Dis. 2025 Jun 23;13(2):101732. doi: 10.1016/j.gendis.2025.101732 (PMC12765100; doi:10.1016/j.gendis.2025.101732)
Supplement: Multimedia component 1 [file mmc1.docx]

**Supplemental materials**

This document contains Figures S1-S9 and Table S1-S4

**Figures and figure legends**

**Figure S1. Integrative bioinformatics analysis for the prostate cancer-specific signature** **screening.** (A-C) Visualization of gene dependency in prostate cancer cell models including DU145, LMCaPclomeFGC, and VCaP with CRISPR-Cas9 screening. (D) Heatmap shows the relative gene expression in prostate cancer compared to normal prostate tissues.

**Figure S2. Integrative analysis revealed a prostate cancer-specific signature comprised of seven genes.** The expression correlation of genes that both dysregulated in prostate cancer and suppressed cell growth after being silenced in different CRPC patient cohorts.

**Figure S3.** Survival analysis of core genes in different prostate cancer patient cohorts.

**Figure S4.** The relative expression of DTL, RRM2, and CDC20 in prostate cancer patients with different Gleason scores in two CRPC patient cohorts.

**Figure S5.** Pearson correlation analysis of DTL, RRM2, and CDC20 with NE score in CRPC cohort.

**Figure S6.** Venn analysis of DTL, RRM2, and CDC20 co-expressed genes in ADPC, CRPC, and NEPC patient cohorts.

**
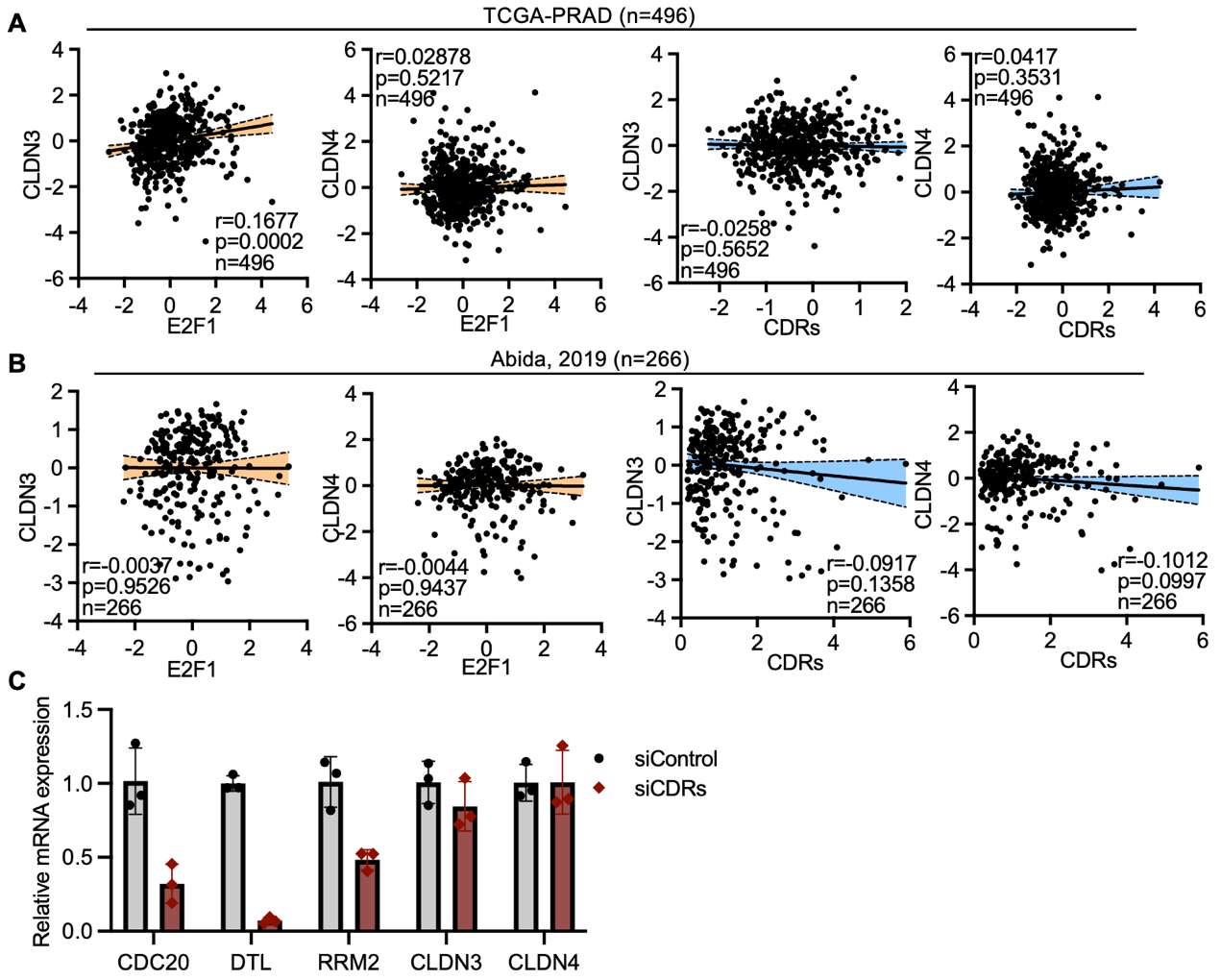
**

**Figure S7. Correlation analysis of CDRs with CLDN3 and CLDN4.** (A and B) Pearson correlation analysis of CDRs or E2F1 with CLDN3 and CLDN4 in different prostate cancer cohorts. (C) The relative expression of CDRs and CLDN3/CLDN4 after CDRs knockdown with CRISPR-Cas13.

**Figure S8.** The relative expression of DTL, RRM2, and CDC20 in CRPC patients with different RB1 deletion status.

**Figure S9.** Molecular docking shows the binding of DTL, RRM2, and CDC20 with Q199, XDD60, and A79.

**Table S1.** gRNA sequence for genes silencing

| **Name** | **Forward 5'-3'** | **Reverse 5'-3'** |
| --- | --- | --- |
| gCDC20-1 | GAACCTTGGAACTGGATTTGCC | GGCAAATCCAGTTCCAAGGTTC |
| gCDC20-2 | GAACTCCAATCCACAAGGTTCA | TGAACCTTGTGGATTGGAGTTC |
| gCDC20-3 | ATTTCGAAGCCGTTTCTGCTGC | GCAGCAGAAACGGCTTCGAAAT |
| gRRM2-1 | GATATCATGGTACTCGATGGGG | CCCCATCGAGTACCATGATATC |
| gRRM2-2 | GAACTTCTTGGCTAAATCGCTC | GAGCGATTTAGCCAAGAAGTTC |
| gRRM2-3 | TTTCTCTTACTCTCTCCTCCGA | CGGAGGAGAGAGTAAGAGAAAT |
| gDTL-1 | GCTACTCGTTACTGTTACTAGG | CCTAGTAACAGTAACGAGTAGC |
| gDTL-2 | GTAACAAGTTTAAGTTCACCAG | CTGGTGAACTTAAACTTGTTAC |
| gDTL-3 | GTGAGCTCCACTGATTTGATTC | GAATCAAATCAGTGGAGCTCAC |
| gRB1 | TTTACTTTGGAAGAGGAAACAA | TTGTTTCCTCTTCCAAAGTAAA |
| gE2F1 | GTTCTTGCTCCAGGCTGAGTAG | CTACTCAGCCTGGAGCAAGAAC |

**Table S2.** Primers used for RT-qPCR

| **Name** | **Forward 5'-3'** | **Reverse 5'-3'** |
| --- | --- | --- |
| qCDC20 | TGGCTGAACTCAAAGGTCACA | CAAAACAGCGCCATAGCCTC |
| qRRM2 | TTACATAAAAGATCCCAAAGAAAGG | AGCCTCTTTGTCCCCAATC |
| qDTL | AACCAAGCACACCATAGCCTTA | GGAGATGGGTAGG GATACAAAC |
| qRB1 | ACTTCTACTCGAACACGAATGC | GTGTCCACCAAGGTCCTGAG |
| qE2F1 | ATGGTGATCAAAGCCCCTCC | AAACATCGATCGGGCCTTGT |
| qCLDN3 | CTGCTCTGCTGCTCGTGTCC | TTAGACGTAGTCCTTGCGGTCGTAG |
| qCLDN4 | TATTGGGGAGGGACGGAAGT | CCTACCCGGAACAGAGGAGA |
| qACTB | ACCGCGAGAAGATGACCCA | GGATAGCACAGCCTGGATAGCAA |

**Table S3.** Primers used for ChIP-qPCR

| **Name** | **Forward 5'-3'** | **Reverse 5'-3'** |
| --- | --- | --- |
| ChIP_DTL-1 | TCTTGGACTCCAGGTTCCCG | GCTCTCTGATTGGCTTCCGA |
| ChIP_DTL-2 | GGGGATCCGAAGGGGGAAAT | AAACTGACGTCACGCTCTCT |
| ChIP_CDC20-1 | TGTTAAAGCCGGTCGGAACT | ACAAAATCAGGGCCACCCTC |
| ChIP_CDC20-2 | GGTTGCGACGGTTGGATTTT | AGTTCCGACCGGCTTTAACA |
| ChIP_RRM2-1 | CCGCGCTGCGCTTGAAAAT | CTGTGCCATGCCTCCGAC |
| ChIP_RRM2-2 | ACTATGCTCTCCCTCCGTGT | GGGCTCACCGTGTTCTCC |

**Table S4.** **Rapid construction of small-molecule library**

| Categories | | | Numbers | Core structure | Representative drugs |
| --- | --- | --- | --- | --- | --- |
| Hydrazones | | | 288 |  |  |
| Olefins   O | Borate alkenes | | 64 |  |  |
|  | Ketenes | | 10 |  |  |
|  | Enols | | 5 |  |  |
| Three- membered ring | | Cyclopropanes | 49 |  |  |
|  |  | Ethylenes | 12 |  |  |
| Ethers | | Ethers | 66 |  |  |
|  |  | Thioethers | 32 |  |  |
| 1,1,-diaryl compounds | | | 33 |  |  |
| Flavanones | | | 83 |  |  |
| Amines | | | 97 |  |  |
| Borides | | | 10 |  |  |
| Fluorenes | | | 62 |  |  |
| Ezetimibe-derivatives | | | 24 |  |  |
| N-heterocycles | | Isoquinoline/ quinoline | 92 |  |  |
|  |  | Triazoles | 32 |  |  |
|  |  | Pyrazoles/Isoxazoles | 59 |  |  |
|  |  | Indoles | 55 |  |  |
|  |  | Pyrimidines | 23 |  |  |
|  |  | 7-membered heterocyclic compounds | 30 |  |  |
|  |  | Luotonin Derivatives | 63 |  |  |
|  |  | Furazans | 31 |  |  |
|  |  | Benzimidazole isoquinoline | 24 |  |  |
| Others | | | 12 | ---- | ------- |
